# Impact of HDL genetic risk scores on coronary artery calcified plaque and mortality in individuals with type 2 diabetes from the Diabetes Heart Study

**DOI:** 10.1186/1475-2840-12-95

**Published:** 2013-06-25

**Authors:** Laura M Raffield, Amanda J Cox, Fang-Chi Hsu, Maggie C-Y Ng, Carl D Langefeld, J Jeffrey Carr, Barry I Freedman, Donald W Bowden

**Affiliations:** 1 Molecular Genetics and Genomics Program, Wake Forest School of Medicine, Winston-Salem, NC, USA; 2 Center for Human Genomics, Wake Forest School of Medicine, Winston-Salem, NC, USA; 3 Center for Diabetes Research, Wake Forest School of Medicine, Winston-Salem, NC, USA; 4 Department of Biochemistry, Wake Forest School of Medicine, Winston-Salem, NC, USA; 5 Department of Biostatistical Sciences, Wake Forest School of Medicine, Winston-Salem, NC, USA; 6 Department of Radiologic Sciences, Wake Forest School of Medicine, Winston-Salem, NC, USA; 7 Department of Internal Medicine - Nephrology, Wake Forest School of Medicine, Winston-Salem, NC, USA

**Keywords:** High-density lipoprotein cholesterol, Type 2 diabetes, Coronary artery calcified plaque, Mortality, Genetic risk score

## Abstract

**Background:**

Patients with type 2 diabetes (T2D) are at elevated risk for cardiovascular disease (CVD) events and mortality. Recent studies have assessed the impact of genetic variants affecting high-density lipoprotein cholesterol (HDL) concentrations on CVD risk in the general population. This study examined the utility of HDL-associated single nucleotide polymorphisms (SNPs) for CVD risk prediction in European Americans with T2D enrolled in the Diabetes Heart Study (DHS).

**Methods:**

Genetic risk scores (GRS) of HDL-associated SNPs were constructed and evaluated for potential associations with mortality and with coronary artery calcified atherosclerotic plaque (CAC), a measure of subclinical CVD strongly associated with CVD events and mortality. Two sets of SNPs were used to construct GRS; while all SNPs were selected primarily for their impacts on HDL, one set of SNPs had pleiotropic effects on other lipid parameters, while the other set lacked effects on low-density lipoprotein cholesterol (LDL) or triglyceride concentrations.

**Results:**

The GRS were specifically associated with HDL concentrations (4.90 × 10^-7^ < p < 0.02) in models adjusted for age, sex, and body mass index (BMI), but were not associated with LDL or triglycerides. Cox proportional hazards regression analysis suggested the HDL-associated GRS had no impact on risk of CVD-mortality (0.48 < p < 0.99) in models adjusted for other known CVD risk factors. However, associations between several of the GRS and CAC were observed (3.85 × 10^-4^ < p < 0.03) in models adjusted for other known CVD risk factors.

**Conclusions:**

The GRS analyzed in this study provide a tool for assessment of HDL-associated SNPs and their impact on CVD risk in T2D. The observed associations between several of the GRS and CAC suggest a potential role for HDL-associated SNPs on subclinical CVD risk in patients with T2D.

## Introduction

Patients with T2D have significantly increased risk for CVD, with mortality rates from heart disease at least two-fold higher than in adults without diabetes [1]. In addition, patients with T2D have higher rates of dyslipidemia, an important CVD risk factor. In particular, individuals with T2D tend to have increased triglyceride concentrations and decreased HDL cholesterol concentrations [2]. An inverse relationship between HDL concentrations and CVD-mortality has long been observed [3]; however, it is not clear whether HDL is a significant contributing factor in development of CVD and whether interventions to specifically alter HDL concentrations markedly impact CVD risk.

Voight et al. [4] used Mendelian randomization to assess the impact of HDL concentrations on myocardial infarction (MI) risk in over 12,000 MI cases and over 40,000 controls. Neither a coding variant in *LIPG* nor a genetic risk score (GRS) derived from 14 common SNPs, both of which were robustly associated with HDL and not with other lipid parameters, was associated with MI. A one standard deviation increase in HDL cholesterol due to GRS was not associated with a significant change in MI risk, though epidemiological data suggested a change in HDL concentration of this magnitude would be associated with an approximate 38% reduction in risk for MI. These data do not support a major role for HDL-associated SNPs in MI.

Given that CVD accounts for more than 65% of all-cause mortality in individuals with T2D, determining the effects of HDL concentrations on CVD in this high risk group is of particular interest [1], as low HDL concentrations have been epidemiologically associated with CVD risk in T2D [5]. Previous large meta-analyses have not specifically analyzed the effect of HDL associated GRS in clinically relevant, community-based cohorts of patients at high risk for CVD, such as patients with T2D. In the current study we constructed GRS containing SNPs associated solely with HDL, SNPs associated with HDL which have apparent pleiotropic effects on other lipid parameters, and both sets of SNPs for analysis in combined scores. We analyzed these GRS for associations with mortality and a measure of subclinical CVD burden, coronary artery calcified atherosclerotic plaque (CAC) [6-8], in a cohort of patients with T2D from the Diabetes Heart Study (DHS).

## Methods

### Study design and sample

DHS participants were recruited from outpatient internal medicine and endocrinology clinics and from the community from 1998 through 2005 in western North Carolina. Siblings concordant for T2D without advanced renal insufficiency were recruited, with additional non-diabetic siblings enrolled whenever possible. Recruitment was based upon family structure, and there were no inclusions/exclusions based on evidence of prevalent CVD at the time of recruitment. Ascertainment and recruitment have been described in detail previously [9-12]. T2D was defined as diabetes developing after the age of 35 years treated with insulin and/or oral agents, in the absence of historical evidence of ketoacidosis. Diabetes diagnosis was confirmed by measurement of fasting glucose and glycated hemoglobin (HbA_1C_) at the exam visit. Analyses completed for the current investigation included 983 self-described European American individuals with T2D from 466 DHS families.

Study protocols were approved by the Institutional Review Board at Wake Forest School of Medicine, and all participants provided written informed consent. Participant examinations were conducted in the General Clinical Research Center of the Wake Forest Baptist Medical Center. Examinations included interviews for medical history and health behaviors, anthropometric measures, resting blood pressure, electrocardiography, fasting blood sampling for laboratory analyses, and spot urine collection. Individuals were considered hypertensive if they were prescribed anti-hypertensive medication or had blood pressure measurements exceeding 140 mmHg (systolic) or 90 mmHg (diastolic). Standard laboratory analyses included fasting glucose, HbA_1C_, total cholesterol, HDL, and triglycerides. Low-density lipoprotein cholesterol (LDL) concentration was calculated using the Friedewald equation, and LDL concentrations were considered valid for subjects whose triglycerides were less than 796 mg/dL. CAC was measured using fast-gated helical computed tomography (CT) scanners, and calcium scores were calculated as previously described and reported as an Agatston score [13,14].

Vital status was determined for all subjects from the National Social Security Death Index maintained by the United States Social Security Administration. For participants confirmed as deceased, length of follow-up was determined from the date of initial study visit to date of death. For all other participants, the length of follow-up was determined from the date of the initial study visit to the end of 2011. For deceased participants, copies of death certificates were obtained from relevant county Vital Records Offices to determine cause of death. Cause of death was categorized based on information contained in death certificates as CVD-mortality (MI, congestive heart failure, cardiac arrhythmia, sudden cardiac death, peripheral vascular disease, and stroke) or either cancer, infection, end-stage renal disease, accidental, or other (including obstructive pulmonary disease, pulmonary fibrosis, liver failure and Alzheimer’s dementia). Association with mortality was assessed for both CVD-mortality and all-cause mortality, i.e. death from any cause.

### Genotyping

Total genomic DNA was purified from whole blood samples using the PUREGENE DNA isolation kit (Gentra Inc., Minneapolis, MN). DNA concentration was quantified using standardized fluorometric readings on a Hoefer DyNA Quant 200 fluorometer (Hoefer Pharmacia Biotech Inc., San Francisco, CA). Genotype data for specific SNPs was derived from: (i) the MassARRAY SNP Genotyping System (Sequenom Inc., San Diego, CA) (n = 4 SNPs), (ii) a genome wide association study (GWAS) using the Affymetrix® Genome-Wide Human SNP Array 5.0 (Affymetrix® Inc., Santa Clara, CA) (n = 2 SNPs), (iii) Illumina® HumanExome BeadChips (Illumina® Inc., San Diego, CA) (n = 18 SNPs), and (iv) GWAS imputed data (n = 4 SNPs).

Genotyping using the MassARRAY SNP Genotyping System was completed as described previously [15]. Primers for PCR amplification and extension reactions were designed using the MassARRAY Assay Design Software (Sequenom). Samples were diluted to a final concentration of 5 ng/μl, and single-base extension reaction products were separated and scored using a matrix-assisted laser desorption ionization/time of flight mass spectrometer. To evaluate genotyping accuracy, 39 quality control samples were included as blind duplicates. The concordance rate for these blind duplicates was 100%.

For the DHS GWAS data, genotype calling was completed using the BRLLM-P algorithm in Genotyping Console v4.0 (Affymetrix). Samples failing to meet an intensity quality control threshold (n = 4) were not included for genotype calling and those failing to meet a minimum acceptable call rate of 95% (n = 3) were excluded from further analyses. An additional 39 samples were included as blind duplicates within the genotyping set to serve as quality controls; the concordance rate for these blind duplicates was 99.0 ± 0.72% (mean ± standard deviation (SD)).

For the DHS Exome Chip data, genotype calling was completed using Genome Studio Software v1.9.4 (Illumina). Samples failing to meet a minimum acceptable call rate of 98% (n = 3) were excluded from further analyses. An additional 58 samples were included as blind duplicates within the genotyping set to serve as quality controls; the concordance rate for blind duplicates was 99.9 ± 0.0001% (mean ± SD). Additional quality control of GWAS and Exome Chip data sets was completed to exclude samples with poor quality genotype calls, gender errors, or unclear/unexpected sibling relationships.

For SNPs where direct genotyping data was not available, genotype data was obtained from GWAS imputed data. Imputation of 1,000 Genomes Project SNPs was completed using the program IMPUTE2 and the Phase I v2, cosmopolitan (integrated) reference panel, build 37 [16,17]. SNPs that were used for imputation were required to have low missingness and show no significant departure from Hardy-Weinberg expectations (p > 1 × 10^-4^). To maximize the quality of imputation, the samples were not pre-phased. Only imputed SNPs with a confidence score > 0.90 and information score > 0.50 were used. A total of ~4.5 million SNPs passed imputation quality control.

For all SNPs used to derive the GRS, the minimum acceptable call rate was 95%; the average SNP call rate was 99.4% ± 1.2% (mean ± SD), and the average sample call rate was 99.4% ± 1.4%. Allele and genotype frequencies were calculated from unrelated individuals and tested for departures from Hardy-Weinberg equilibrium. No SNPs showed significant departure from Hardy-Weinberg equilibrium (p > 0.05). One SNP (rs386000) included by Voight et al. [4] in their HDL GRS failed genotyping and was not included in the current analysis.

### GRS calculation

Both unweighted GRS and GRS weighted by SNP effect size were derived for two sets of SNPs previously reported to be associated with HDL [4]. One set of 14 SNPs had documented effects on HDL concentrations only and were used by Voight et al. [4] for construction of a GRS. We created GRS from 13 of these SNPS with good quality genotyping data (rs386000 excluded) (Score 1; 1a = unweighted, 1b = weighted).

In addition, Voight et al. [4] also reported an additional set of 15 SNPs, including a coding variant in *LIPG*, as associated with HDL concentrations, with some of these SNPs also reported to have pleiotropic effects on LDL cholesterol and triglyceride concentrations. All SNPs were primarily selected for their impact on HDL. Weighted and unweighted GRS were derived from this additional set of 15 SNPs (Score 2; 2a = unweighted, 2b = weighted). SNPs (n = 26) from both sets were also combined to derive unweighted and weighted combined GRS; two pairs of SNPs (rs2338104 and rs7134594; rs2271293 and rs16942887) are in strong linkage disequilibrium (r^2^ > 0.90), and as such two SNPs (rs7134594 and rs2271293) were excluded from the combined scores. SNPs in the combined GRS were weighted by their effect sizes in mmol/L. The effect sizes used were drawn from the Voight et al. paper or the Global Lipid Genetic Consortium GWAS for Lipids paper that Voight et al. cited [4,18]. All derived GRS (1a, 1b, 2a, 2b, Combined Unweighted, Combined Weighted) were tested for association with HDL, LDL and triglycerides to evaluate whether the GRS were a measure of genetic contributions to either HDL only or to global lipid levels.

For all GRS, the effect allele was assigned as the allele associated with an increase in plasma HDL concentrations, i.e., an increase in GRS can be interpreted as an increase in genetic predisposition for elevated plasma HDL, as seen in the GRS used by Voight et al. [4]. Unweighted scores were derived by adding the number of effect alleles across each SNP. The SNPs were also weighted by their previously reported effect sizes [4]. For the weighted scores, the number of effect alleles possessed by an individual at a particular SNP locus was multiplied by a weight derived from that SNP’s effect size contribution to the total effect size for all SNPs included in the GRS. For individuals missing genotype data for a particular SNP, the mean genotype calculated in the DHS for that given SNP was assigned [19].

### Statistical analysis

For statistical analyses, continuous variables were transformed as necessary to approximate normality. Single SNP association analyses were performed using variance components methods implemented in Sequential Oligogenic Linkage Analysis Routines (SOLAR) version 6.4.1 (Texas Biomedical Research Institute, San Antonio, TX) to account for relatedness between subjects [20]. Association was examined assuming an additive model of inheritance. Age and sex were included as covariates in single SNP association analyses for HDL.

GRS were considered as both ordinal (three tertiles: T1, T2, T3 derived from increasing tertile ranges) and continuous variables. Relationships between the GRS and HDL, LDL, triglycerides, CAC, and prior history of CVD and MI were examined using marginal models with generalized estimating equations. The models account for familial correlation using a sandwich estimator of the variance under exchangeable correlation. Relationships between GRS and both all-cause and CVD-mortality were examined using Cox proportional hazards models with sandwich-based variance estimation due to the inclusion of related individuals in this study. Associations were adjusted for covariates including age, sex, BMI, smoking status (history of current or prior smoking), hypertension, cholesterol medication use, prior CVD, oral T2D medication use, and insulin use as indicated. All analyses were performed in SAS 9.3 (SAS Institute, Cary, NC). Statistical significance was accepted at p < 0.05.

## Results

The goal of this study was to assess the impact of HDL-associated SNPs on CVD risk in subjects from the DHS of families enriched for T2D. We assessed the relationships between GRS based on HDL-associated SNPs and both subclinical CVD and mortality risk. The clinical characteristics of the 983 European American individuals with T2D included in the study are summarized in Table 1. Subjects exhibited a variety of known CVD risk factors, including hypertension, high body mass, and dyslipidemia. Subclinical CVD was prevalent, demonstrated by the large number of subjects who had detectable CAC.

**Table 1:** Demographic and clinical characteristics of 983 individuals with type 2 diabetes from the Diabetes Heart Study

	**Mean ± SD or %**	**Median (range)**
**Demographic information**		
Age (years)	62.5 ± 9.1	63 (34.2-86)
Gender (% female)	51.8%	
T2D Duration (years)	10.5 ± 7.2	8 (0-46)
Smoking (current or past) (%)	59.4%	
Self-reported history of prior CVD (%)	43.3%	
Deceased (%)	23.3%	
Deceased (from CVD) (%)	10.3%	
**Body composition**		
Height (cm)	168.7 ± 9.7	168.5 (122.8-202.0)
Weight (kg)	92.2 ± 20.3	89.5 (40.8-209.2)
BMI (kg/m^2^)	32.3 ± 6.6	31.2 (17.1-58.0)
**Medications**		
Cholesterol Medications (%)	47.8%	
Oral T2D Medications (%)	78.8%	
Insulin (%)	27.6%	
**Blood pressure**		
Systolic BP (mmHg)	140 ± 18.9	138.5 (94-260)
Diastolic BP (mmHg)	72.7 ± 10.3	72 (36.5-106)
Hypertension (%)	88.9%	
**Blood biochemistry**		
Glucose (mg/dL)	148.1 ± 56.2	135 (16-463)
Hemoglobin A_1C_ (%)	7.6 ± 1.7	7.2 (4.3-18.3)
Total Cholesterol (mg/dL)	184.9 ± 43.7	180.5 (65-427)
HDL cholesterol (mg/dL)	42.2 ± 12	41 (8-98)
LDL cholesterol (mg/dL)	102.8 ± 32.6	100 (12-236)
Triglycerides (mg/dL)	208.8 ± 140.2	175 (30-1310)
**Vascular imaging**		
Coronary Artery Calcified Plaque (CAC)	1888 ± 3382	478 (0-50415)

A total of 28 SNPs were genotyped (Table 2), including 13 SNPs solely associated with HDL and 15 SNPs associated with HDL that may also have pleiotropic effects on other lipid parameters [4]. We first performed single SNP association analyses for these 28 SNPs included in our GRS. Four SNPs (rs3764261, rs1800588, rs3890182, rs4759375) were associated (7.00 × 10^-5^ < p < 0.02) with HDL concentrations, with the estimated effects in the same direction and of similar magnitude as those previously reported by Voight et al. [4]. The coding variant in *LIPG* (rs61755018) reported by Voight et al. was not significantly associated with HDL concentrations in our cohort [4].

**Table 2:** HDL-associated SNPs included in the genetic risk scores (GRS)

		**SNPs in risk scores 1a and 1b**						
**SNP**	**Chr**	**Position**	**Gene(s)**	**Source**	**Alleles (Major/Minor)**	**Effect Allele**	**Effect Allele Frequency DHS**	**Effect (βvalue) in mmol/L**
rs1689800	1	182168885	GLUL, ZNF648	Exome Chip	A/G	A	0.628	0.01
rs13107325	4	103188709	SLC39A8	Exome Chip	C/T	C	0.925	0.02
rs2293889	8	116599199	TRPS1	Sequenom	G/T	G	0.554	0.01
rs2923084	11	10388782	AMPD3	Exome Chip	A/G	A	0.815	0.01
rs7134594	12	110000193	MMAB	Exome Chip	T/C	T	0.520	0.01
rs4759375	12	123796238	SBNO1	Imputed data	C/T	T	0.092	0.02
rs838880	12	125261593	SCARB1	Exome Chip	T/C	C	0.337	0.02
rs16942887	16	67928042	PSKH1	Exome Chip	G/A	A	0.115	0.03
rs881844	17	37810218	STARD3	Imputed data	G/C	G	0.652	0.01
rs4082919	17	76377482	PGS1	Sequenom	G/T	T	0.483	0.01
rs7255436	19	8433196	ANGPTL4	Exome Chip	A/C	A	0.506	0.01
rs737337	19	11347493	DOCK6	Exome Chip	T/C	T	0.900	0.02
rs181362	22	21932068	UBE2L3	Sequenom	C/T	C	0.813	0.01
		**SNPs in risk scores 2a and 2b**						
rs4846914	1	230295691	GALNT2	Sequenom	A/G	A	0.606	0.02
rs17145738	7	72982874	TBL2, BCL7B	Exome Chip	C/T	T	0.124	0.03
rs17482753	8	19832646	LPL	Exome Chip	G/T	T	0.095	0.08
rs17321515	8	126486409	TRIB1	GWAS	A/G	G	0.465	0.02
rs471364	9	15289578	TTC39B	Exome Chip	T/C	T	0.896	0.03
rs3890182	9	107647655	ABCA1	Exome Chip	G/A	G	0.890	0.03
rs174547	11	61570783	FADS1	Exome Chip	T/C	T	0.668	0.03
rs6589566	11	116652423	ZNF259, APOA5	GWAS	A/G	A	0.924	0.05
rs2338104	12	109895168	KCTD10	Exome Chip	G/C	G	0.520	0.03
rs1800588	15	58723675	LIPC	Exome Chip	C/T	T	0.207	0.05
rs3764261	16	56993324	CETP	Exome Chip	C/A	A	0.331	0.1
rs2271293	16	67902070	NUTF2	Exome Chip	G/A	A	0.113	0.03
rs61755018	18	47109955	LIPG	Imputed data	A/G	G	0.007	0.14
rs2967605	19	8469738	RAB11B	Exome Chip	C/T	C	0.812	0.05
rs16988929	20	42904315	GDAP1L1	Imputed data	C/T	T	0.002	0.01

SNPs in Risk Score 1a (unweighted) and Risk Score 1b (weighted) have effects on HDL levels only, while SNPs in Risk Score 2a (unweighted) and Risk Score 2b (weighted) have effects on HDL levels as well as some pleiotropic effects on LDL and triglyceride levels. Allele frequencies in the DHS are provided along with the effect size estimates (β value) reported by Voight et al.

Next, weighted and unweighted GRS were analyzed. One GRS was derived from 13 SNPs (rs386000 excluded) reported to have effects solely on HDL (Score 1; 1a = unweighted, 1b = weighted; Table 2); scores ranged from 8.0 to 21.0 (14.7 ± 2.2, mean ± SD) for GRS 1a and from 7.8 to 21.0 (13.4 ± 2.1) for GRS 1b. The second GRS was derived from an additional set of 15 SNPs associated with HDL concentrations, some of which also have pleiotropic effects on LDL cholesterol and triglycerides (Score 2; 2a = unweighted, 2b = weighted; Table 2); scores ranged from 7.0 to 20.0 (13.3 ± 2.0) for GRS 2a and from 5.4 to 17.8 (11.0 ± 2.1) for GRS 2b. Additional GRS were constructed from a combined set of 26 SNPs, with two SNPs not included in the combined scores due to strong linkage disequilibrium with other included SNPs (Combined Unweighted and Combined Weighted scores; Table 2); scores ranged from 17.0 to 36.0 (26.8 ± 2.9) for the Combined Unweighted GRS and from 12.2 to 31.8 (21.2 ± 3.2) for the Combined Weighted GRS.

An initial analysis of tertiles of GRS 1 and 2 (i.e. upper, middle, and lower third of GRS distribution) as an ordinal variable was performed to test for a linear trend of association with plasma HDL concentrations. As GRS 1 and 2 were linearly associated with HDL concentrations (6.18 × 10^-6^ < p < 0.007 in models adjusted for additional CVD risk factors), we next examined the GRS as continuous variables, including GRS 1 and 2 and the Combined GRS. When analyses were adjusted for age, sex, and BMI, all weighted and unweighted GRS were associated with increased HDL (Table 3) but were not associated with LDL cholesterol or triglyceride concentrations (p > 0.05). In addition, significant associations were observed between several of the GRS and CAC (Table 3). This suggests a potential impact of a genetic predisposition to increased HDL on reduced risk for subclinical CVD in patients with T2D. Further analyses indicated that the GRS were not significantly associated with CVD-mortality (Table 4), nor were any significant associations observed between the GRS and history of prior CVD or history of prior MI (0.06 < p < 0.87). The GRS were also not associated with all-cause mortality, with the exception of the nominal associations of GRS 2b, the weighted GRS which includes some SNPs with pleiotropic effects on lipid parameters other than HDL, and the Combined Weighted GRS (Table 4). Analyses were also performed with further adjustment for diabetic medication use; results were essentially unchanged (Additional files 1 and 2).

**Table 3:** Associations between HDL genetic risk scores and HDL, LDL, triglycerides, and coronary artery calcified plaque (CAC)

	**Model 1**		**Model 2**		**Model 3**	
	**β Estimate (CI)**	**p-value**	**β Estimate (CI)**	**p-value**	**β Estimate (CI)**	**p-value**
**HDL**						
Risk score 1a	0.027 (-0.001- 0.055)	0.057	0.030 (0.005- 0.056)	0.022	0.033 (0.007- 0.058)	0.013
Risk score 1b	0.033 (0.003- 0.063)	0.031	0.034 (0.006- 0.061)	0.018	0.035 (0.008- 0.063)	0.012
Risk score 2a	0.046 (0.016- 0.076)	0.003	0.051 (0.023- 0.079)	3.85 × 10^-4^	0.052 (0.024- 0.080)	3.06 × 10^-4^
Risk score 2b	0.061 (0.034- 0.089)	1.42 × 10^-5^	0.062 (0.036- 0.088)	2.60 × 10^-6^	0.063 (0.037- 0.088)	1.44 × 10^-6^
Combined unweighted	0.035 (0.015- 0.055)	0.001	0.039 (0.021- 0.058)	3.63 × 10^-5^	0.040 (0.021- 0.058)	2.34 × 10^-5^
Combined weighted	0.043 (0.025- 0.061)	4.65 × 10^-6^	0.044 (0.027- 0.061)	4.90 × 10^-7^	0.044 (0.027- 0.061)	2.75 × 10^-7^
**LDL**						
Risk score 1a	0.317 (-0.678- 1.311)	0.532	0.260 (-0.74- 1.260)	0.611	0.245 (-0.759- 1.248)	0.633
Risk score 1b	0.243 (-0.782- 1.268)	0.642	0.177 (-0.854- 1.207)	0.737	0.140 (-0.887- 1.167)	0.789
Risk score 2a	−0.823 (-1.978- 0.332)	0.163	−0.838 (-1.987- 0.312)	0.153	−0.805 (-1.955- 0.346)	0.170
Risk score 2b	−0.507 (-1.614- 0.600)	0.369	−0.604 (-1.713- 0.506)	0.286	−0.585 (-1.698- 0.529)	0.303
Combined unweighted	−0.243 (-1.018- 0.533)	0.539	−0.261 (-1.033- 0.512)	0.509	−0.266 (-1.038- 0.507)	0.501
Combined weighted	−0.260 (-0.988- 0.468)	0.484	−0.325 (-1.054- 0.404)	0.383	−0.327 (-1.060- 0.406)	0.381
**Triglycerides**						
Risk score 1a	−0.002 (-0.019- 0.015)	0.853	−0.005 (-0.021- 0.012)	0.591	−0.006 (-0.022- 0.010)	0.469
Risk score 1b	−0.005 (-0.022- 0.013)	0.595	−0.007 (-0.024- 0.010)	0.402	−0.008 (-0.024- 0.009)	0.374
Risk score 2a	−0.013 (-0.034- 0.008)	0.220	−0.016 (-0.036- 0.004)	0.123	−0.015 (-0.036- 0.005)	0.133
Risk score 2b	−0.009 (-0.028- 0.010)	0.370	−0.013 (-0.031- 0.005)	0.162	−0.013 (-0.031- 0.005)	0.163
Combined unweighted	−0.010 (-0.023- 0.004)	0.157	−0.012 (-0.025- 0.001)	0.076	−0.012 (-0.025- 0.001)	0.072
Combined weighted	−0.007 (-0.020- 0.005)	0.246	−0.010 (-0.022- 0.002)	0.092	−0.010 (-0.022- 0.002)	0.096
**Coronary Artery Calcified Plaque (CAC)**						
Risk score 1a	−0.079 (-0.154- -0.003)	0.042	−0.065 (-0.128- -0.001)	0.046	−0.068 (-0.128- -0.008)	0.027
Risk score 1b	−0.067 (-0.146- 0.012)	0.097	−0.053 (-0.119- 0.014)	0.119	−0.059 (-0.123- 0.004)	0.065
Risk score 2a	−0.072 (-0.154- 0.011)	0.089	−0.063 (-0.134- 0.009)	0.085	−0.082 (-0.149- -0.014)	0.017
Risk score 2b	−0.117 (-0.197- -0.036)	0.005	−0.088 (-0.156- -0.020)	0.011	−0.101 (-0.163- -0.038)	0.002
Combined unweighted	−0.083 (-0.140- -0.026)	0.004	−0.077 (-0.123- -0.031)	0.001	−0.080 (-0.124- -0.037)	3.18 × 10^-4^
Combined weighted	−0.087 (-0.141- -0.033)	0.002	−0.069 (-0.114- -0.024)	0.002	−0.075 (-0.117- -0.034)	3.85 × 10^-4^

Analysis was performed using marginal models with generalized estimating equations. Model 1 is unadjusted; Model 2 is adjusted for age, sex, and body mass index (BMI); Model 3 is adjusted for age, sex, BMI, smoking, hypertension, and prior cardiovascular disease. Associations are reported as the β estimate and its 95% confidence interval (CI).

**Table 4:** Association between HDL genetic risk scores analyzed as a continuous variable and all-cause and CVD-mortality

	**Model 1**		**Model 2**		**Model 3**	
**All-cause mortality**	**HR (CI)**	**p-value**	**HR (CI)**	**p-value**	**HR (CI)**	**p-value**
Risk score 1a	0.96 (0.91, 1.02)	0.208	0.98 (0.92, 1.03)	0.373	0.97 (0.92, 1.03)	0.295
Risk score 1b	0.96 (0.91, 1.02)	0.175	0.97 (0.92, 1.03)	0.314	0.97 (0.92, 1.02)	0.228
Risk score 2a	0.96 (0.90, 1.03)	0.235	0.97 (0.91, 1.03)	0.302	0.96 (0.90, 1.03)	0.232
Risk score 2b	0.92 (0.87, 0.98)	0.010	0.93 (0.88, 0.99)	0.020	0.93 (0.88, 0.98)	0.011
Combined unweighted	0.96 (0.92- 1.01)	0.115	0.97 (0.93- 1.01)	0.170	0.97 (0.93- 1.01)	0.162
Combined weighted	0.95 (0.91- 0.99)	0.009	0.96 (0.92- 0.99)	0.017	0.95 (0.92- 0.99)	0.011
**CVD mortality**						
Risk score 1a	1.00 (0.92, 1.09)	0.958	1.01 (0.93, 1.10)	0.771	1.00 (0.92, 1.09)	0.988
Risk score 1b	1.00 (0.92, 1.09)	0.936	1.01 (0.93, 1.10)	0.880	0.99 (0.91, 1.08)	0.835
Risk score 2a	1.03 (0.93, 1.13)	0.621	1.03 (0.94, 1.13)	0.542	1.02 (0.94, 1.12)	0.622
Risk score 2b	0.96 (0.89, 1.05)	0.407	0.98 (0.90, 1.06)	0.547	0.97 (0.90, 1.05)	0.477
Combined unweighted	1.00 (0.94- 1.07)	0.955	1.01 (0.95- 1.07)	0.826	1.00 (0.94- 1.07)	0.890
Combined weighted	0.98 (0.92- 1.04)	0.429	0.98 (0.93- 1.04)	0.573	0.98 (0.93- 1.04)	0.502

Analysis was performed using Cox proportional hazards regression. Model 1 is unadjusted; Model 2 is adjusted for age and sex; Model 3 is adjusted for age, sex, body mass index, smoking, hypertension, cholesterol medication use, and prior cardiovascular disease. The hazard ratio (HR) and its 95% confidence interval (CI) are reported.

In an effort to further quantify these findings, tertiles of GRS 1 and 2 were also analyzed in reference to the lowest GRS tertile. The highest tertile was consistently associated with increased HDL concentrations (Additional file 3), with GRS 2b displaying the strongest association (p = 4.31 × 10^-5^ for comparison of the highest in reference to the lowest GRS tertile). Further quantification of risk for all-cause mortality suggests individuals in the highest tertile for GRS 2b experience an approximate 40% reduction in risk of mortality compared to those in the lowest tertile (HR: 0.57; 95% CI: 0.39- 0.83; p = 0.003).

## Discussion

The present study evaluated HDL-associated SNPs in patients with T2D using weighted and unweighted GRS. It is important to assess the impact of HDL-associated SNPs in patients with T2D, as decreased HDL concentrations are epidemiologically associated with higher CVD-mortality and are often observed in patients with T2D [2,3]. In addition to conventional lipid parameters, measures of prevalent disease, and mortality, we evaluated associations of these GRS with CAC. CAC has been shown in the DHS and other cohorts to be a strong independent predictor of CVD events and mortality [6,8]. Compared to individuals with low or nonexistent CAC (CAC 0–9), individuals in the DHS with very high CAC (≥ 1,000) experienced >6-fold increased risk for all-cause mortality and >11-fold increased risk for CVD-mortality [8,21]. CAC has not previously been examined in studies of the impact of HDL-associated variants on CVD risk. Hence, analysis of our HDL GRS with this subclinical measure of CVD was unique and of clinical relevance.

For the weighted and unweighted GRS assessed, higher GRS values were clearly associated with increased plasma HDL. The GRS were not associated with LDL cholesterol and triglyceride concentrations, allowing assessment of the effects of genetic predisposition to higher or lower HDL without confounding effects from associations with other lipid parameters. No evidence of association between the GRS and CVD-mortality was observed; however, associations between the GRS and CAC were observed. These associations point to a potential reduction in risk for subclinical CVD in patients with T2D with a genetic predisposition to increased HDL.

### Prior analyses of HDL-associated SNPs

Recently, Voight et al. [4] evaluated two sets of SNPs associated with higher HDL to determine whether HDL-associated SNPs would confer protection from MI in the general population. They concluded that a genetic predisposition to higher HDL did not detectably influence MI risk. However, Voight et al. did not address the association of their GRS in high CVD risk groups such as patients with T2D [4]. Moreover, it is important to test the applicability of results from extremely large meta-analyses in community-based cohorts. This is a necessary step in assessing the value of these GRS for introduction into clinical medicine. We hypothesized that GRS of HDL-associated SNPs could provide a tool for evaluating the effect of HDL concentrations on CVD risk in patients with T2D.

Prior studies in the general population have generally shown little impact of genetic variants affecting HDL concentrations on CVD risk. In two large Danish studies, variants associated with a decrease in HDL cholesterol did not increase CVD risk [22,23]. In another study with 8,473 Caucasian participants, GRS were strongly associated with HDL concentrations, but were not associated with CVD [24]. However, few previous studies have focused on patients with T2D, so it is unclear whether HDL-associated SNPs impact CVD risk in this group, a gap that we addressed in this study.

### Analysis of GRS for associations with lipid parameters

We did not detect many of the single SNP associations with HDL concentrations reported by Voight et al. [4]. This is not surprising, as the current study has lower power to detect associations given the modest effect sizes previously reported. However, we observed significant associations between all of the GRS assessed and HDL, allowing us to consider the impact of genetic determinants of HDL concentrations on CVD risk. Some past studies of SNPs associated with HDL in patients with T2D have examined SNPs with pleiotropic effects on other lipid parameters or other traits, such as obesity, which could also influence CVD risk [25-28]. An important strength of this study is that the GRS were not associated with LDL cholesterol and triglyceride concentrations, indicating the GRS are mainly informative for an individual’s HDL concentration, as opposed to more global lipid concentrations.

In our study, consistent with the GRS used by Voight et al., a higher GRS indicates a predisposition to higher HDL [4]. While we refer to all GRS as risk scores by convention, epidemiological data would in fact indicate that higher GRS values should be associated with increased protection from CVD, not increased risk, as higher plasma HDL is protective [3]. However, constructing the GRS so that higher GRS values would be associated with decreased HDL would not have changed our results; p-values would remain unchanged and effect estimates would be of the same magnitude in the opposite direction.

### Analysis of GRS for associations with mortality and subclinical CVD

Association of the GRS with HDL allowed us to subsequently consider the impact of genetic predisposition to higher or lower HDL on all-cause mortality and CVD-mortality in T2D. While the HDL GRS were not associated with CVD-mortality, association with all-cause mortality was observed for GRS 2b, the weighted GRS which includes SNPs with pleiotropic effects on LDL cholesterol and triglycerides, and the Combined Weighted GRS. It is possible that these associations are due to the reported pleiotropic effects on LDL and triglycerides of some of the included SNPs, although the GRS overall were not associated with LDL and triglyceride concentrations. Alternatively this observation may be due to impacts of HDL on risk for mortality through pathways other than CVD. A recent study by Qi et al. [29] examined HDL, LDL, and triglyceride GRS and demonstrated a modest increase in T2D risk with increased HDL GRS. While the current study consists entirely of T2D affected individuals, the study by Qi et al. [29] suggests a role for HDL in diabetes pathogenesis, perhaps impacting mortality. Apart from reverse cholesterol transport, the cardinal function of HDL in ameliorating CVD risk, HDL particles also demonstrate anti-inflammatory, antioxidant, and anti-apoptotic effects which may be important mechanisms contributing to risk for mortality [30]. Further, while the GRS assessed in this study are associated with HDL concentrations, their impact on HDL particle composition and function is unclear and may further contribute to mechanisms underpinning the observed associations with all-cause mortality.

While we did not observed significant associations between the HDL GRS and CVD-mortality, we did observe interesting associations between the GRS and CAC, a measure of subclinical CVD risk. We observed an association between GRS 1a, the unweighted GRS which contains SNPs which affect HDL concentrations only, and CAC. Likewise associations with CAC were observed for GRS 2a and 2b (SNPs with pleiotropic effects on lipid parameters other than HDL), as well as the Combined Unweighted and Weighted GRS. This points to a potential role of HDL-associated SNPs in subclinical CVD risk; it is possible we are not observing the impact of HDL-associated SNPs on CVD-mortality due to a low number of events, with association with CAC observed due to increased power for analyses of this continuous trait. This may point to a differing impact of HDL-associated SNPs in individuals with T2D than is seen in most studies of the general population. However, little work on associations between CAC and HDL-associated SNPs has been done in the general population, so these SNPs may be associated with CAC in the general population as well.

### Limitations

SNPs included in our GRS were selected for the strength of their association with HDL concentrations in previous studies [4,18]; other SNPs more modestly associated with HDL were not the focus of the current study but may, in some cases, have a greater impact on disease risk [27,31]. The impact of genetic variants associated with HDL may also be modulated by environmental factors, which was not assessed here [32,33]. The disparate treatments prescribed to individuals in the DHS, including oral T2D medications and insulin, may also impact CVD outcomes; however, inclusion of these covariates in our models did not substantially change our results (Additional files 1 and 2). For analyses of CVD mortality, our power to detect a modest effect (HR ~ 1.1) associated with our GRS was limited (<60% power); however, our power to detect a larger effect (HR ~ 1.2) was high (>95% power). As a result, we cannot exclude a modest impact of our HDL GRS on CVD mortality that was not detected in this study. In contrast, limited power was not a concern for analyses of all-cause mortality (>85% power to detect a HR of ~1.1) due to the increased number of events, nor for analyses of continuous measures such as CAC and HDL (>85% power for a modest effect size of 0.02).

## Conclusions

In summary, the GRS analyzed in this paper provide a useful tool for assessing the impact of genetic predisposition to higher or lower HDL concentrations on risk for CVD in patients with T2D. As has been observed in studies in the general population, there was no association between the HDL GRS and risk of CVD-mortality. However, associations between some of the GRS and CAC were observed, pointing to a potential role of genetic variants affecting HDL concentrations in risk for subclinical CVD in patients with T2D. Further study of HDL-associated genetic variants will be needed to further clarify whether these variants are important determinants of CVD risk in T2D affected individuals.

## Abbreviations

BMI: Body mass index; CAC: Coronary artery calcified plaque; CT: Computed tomography; CVD: Cardiovascular disease; DHS: Diabetes Heart Study; HDL: High-density lipoprotein cholesterol; GRS: Genetic risk score; GWAS: Genome wide association study; HbA1C: Glycated hemoglobin; LDL: Low-density lipoprotein cholesterol; MI: Myocardial infarction; SD: Standard deviation; SNP: Single nucleotide polymorphism; SOLAR: Sequential Oligogenic Linkage Analysis Routines; T2D: Type 2 diabetes.

## Competing interests

The authors declare that they have no competing interests.

## Authors’ contributions

LMR performed SNP genotyping, perfomed statistical analysis, and prepared the manuscript; AJC assisted with SNP genotyping, contributed to the statistical analysis, and assisted with the manuscript preparation; FCH contributed to the statistical analysis and reviewed and edited the manuscript; MCYN contributed to the management of the genetic data and reviewed the manuscript; CDL performed the SNP imputation and reviewed the manuscript; JJC was involved in the initial design of the Diabetes Heart Study, contributed to patient ascertainment and clinical evaluation, and reviewed the manuscript; BIF was involved in the initial design of the Diabetes Heart Study, contributed to patient ascertainment and clinical evaluation, and reviewed and edited the manuscript; DWB leads the Diabetes Heart Study and assisted with the manuscript preparation. All authors read and approved the final manuscript.

## Supplementary Material

Additional file 1 Associations between HDL genetic risk scores and HDL, LDL, triglycerides, and coronary artery calcified plaque (CAC), with and without adjustment for diabetic medication use. Click here for file

Additional file 2 Association between HDL genetic risk scores analyzed as a continuous variable and all-cause and CVD-mortality, with and without adjustment for diabetic medication use. Click here for file

Additional file 3 **Association between HDL genetic risk score tertiles and all-cause and CVD- mortality using unadjusted proportional hazards regression models and between risk scores and HDL levels using unadjusted marginal models incorporating generalized estimating equations.** Hazard ratios (HR) or β estimates (as appropriate) and 95% confidence intervals (CI) are reported relative to the lowest tertile. Click here for file
