# Supplementary material for: Impact of HDL genetic risk scores on coronary artery calcified plaque and mortality in individuals with type 2 diabetes from the Diabetes Heart Study
Source: Cardiovasc Diabetol. 2013 Jun 25;12:95. doi: 10.1186/1475-2840-12-95 (PMC3695806; doi:10.1186/1475-2840-12-95)
Supplement: Additional file 1 — Associations between HDL genetic risk scores and HDL, LDL, triglycerides, and coronary artery calcified plaque (CAC), with and without adjustment for diabetic medication use. [file 1475-2840-12-95-S1.pdf]

Additional file 1: Associations between HDL genetic risk scores and HDL, LDL, triglycerides, and coronary artery calcified plaque (CAC), with and without adjustment for diabetic medication use.

	Model 1		Model 2	
	**β Estimate (CI)**	**p-value**	**β Estimate (CI)**	**p-value**
**HDL**				
Risk Score 1a	0.033 (0.007- 0.058)	0.013	0.034 (0.008- 0.059)	0.009
Risk Score 1b	0.035 (0.008- 0.063)	0.012	0.036 (0.009- 0.063)	0.010
Risk Score 2a	0.052 (0.024- 0.080)	3.06×10^{-4}	0.051 (0.023- 0.079)	4.00×10^{-4}
Risk Score 2b	0.063 (0.037- 0.088)	1.44×10^{-6}	0.062 (0.036- 0.088)	2.14×10^{-6}
Combined Unweighted	0.040 (0.021- 0.058)	2.34×10^{-5}	0.040 (0.022- 0.059)	1.49×10^{-5}
Combined Weighted	0.044 (0.027- 0.061)	2.75×10^{-7}	0.044 (0.027- 0.061)	3.40×10^{-7}
**LDL**				
Risk Score 1a	0.245 (-0.759- 1.248)	0.633	0.301 (-0.695- 1.297)	0.554
Risk Score 1b	0.140 (-0.887- 1.167)	0.789	0.166 (-0.850- 1.181)	0.749
Risk Score 2a	-0.805 (-1.955- 0.346)	0.170	-0.841 (-1.977- 0.296)	0.147
Risk Score 2b	-0.585 (-1.698- 0.529)	0.303	-0.612 (-1.719- 0.495)	0.279
Combined Unweighted	-0.266 (-1.038- 0.507)	0.501	-0.238 (-1.007- 0.531)	0.545
Combined Weighted	-0.327 (-1.060- 0.406)	0.381	-0.334 (-1.063- 0.395)	0.369
**Triglycerides**				

Risk Score 1a	-0.006 (-0.022- 0.010)	0.469	-0.007 (-0.023- 0.010)	0.432
Risk Score 1b	-0.008 (-0.024- 0.009)	0.374	-0.008 (-0.024- 0.009)	0.357
Risk Score 2a	-0.015 (-0.036- 0.005)	0.133	-0.016 (-0.036- 0.005)	0.132
Risk Score 2b	-0.013 (-0.031- 0.005)	0.163	-0.013 (-0.031- 0.005)	0.158
Combined Unweighted	-0.012 (-0.025- 0.001)	0.072	-0.012 (-0.025- 0.001)	0.063
Combined Weighted	-0.010 (-0.022- 0.002)	0.096	-0.010 (-0.022- 0.002)	0.090
**Coronary Artery Calcified Plaque**				
Risk Score 1a	-0.068 (-0.128- -0.008)	0.027	-0.066 (-0.127- -0.006)	0.032
Risk Score 1b	-0.059 (-0.123- 0.004)	0.065	-0.058 (-0.122- 0.005)	0.071
Risk Score 2a	-0.082 (-0.149- -0.014)	0.017	-0.080 (-0.147- -0.013)	0.019
Risk Score 2b	-0.101 (-0.163- -0.038)	0.002	-0.099 (-0.162- -0.037)	0.002
Combined Unweighted	-0.080 (-0.124- -0.037)	3.18×10^{-4}	-0.079 (-0.123- -0.035)	4.32×10^{-4}
Combined Weighted	-0.075 (-0.117- -0.034)	3.85×10^{-4}	-0.074 (-0.115- -0.032)	4.83×10^{-4}

Analysis was performed using marginal models with generalized estimating equations. Model 1 is adjusted for age, sex, body mass index (BMI), smoking, hypertension, and prior cardiovascular disease; Model 2 is adjusted for age, sex, BMI, smoking, hypertension, prior cardiovascular disease, oral T2D medication use, and insulin use. Associations are reported as the β estimate and its 95% confidence interval (CI).
