# Supplementary material for: Impact of HDL genetic risk scores on coronary artery calcified plaque and mortality in individuals with type 2 diabetes from the Diabetes Heart Study
Source: Cardiovasc Diabetol. 2013 Jun 25;12:95. doi: 10.1186/1475-2840-12-95 (PMC3695806; doi:10.1186/1475-2840-12-95)
Supplement: Additional file 2 — Association between HDL genetic risk scores analyzed as a continuous variable and all-cause and CVD-mortality, with and without adjustment for diabetic medication use. [file 1475-2840-12-95-S2.pdf]

Additional file 2: Association between HDL genetic risk scores analyzed as a continuous variable and all-cause and CVD-mortality, with and without adjustment for diabetic medication use.

	Model 1		Model 2	
**All-cause Mortality**	**HR (CI)**	**p-value**	**HR (CI)**	**p-value**
Risk Score 1a	0.97 (0.92, 1.03)	0.295	0.97 (0.92, 1.03)	0.332
Risk Score 1b	0.97 (0.92, 1.02)	0.228	0.97 (0.92, 1.02)	0.259
Risk Score 2a	0.96 (0.90, 1.03)	0.232	0.96 (0.91, 1.03)	0.243
Risk Score 2b	0.93 (0.88, 0.98)	0.011	0.93 (0.88, 0.99)	0.014
Combined Unweighted	0.97 (0.93- 1.01)	0.162	0.97 (0.93- 1.01)	0.185
Combined Weighted	0.95 (0.92- 0.99)	0.011	0.96 (0.92- 0.99)	0.016
**CVD Mortality**				
Risk Score 1a	1.00 (0.92, 1.09)	0.988	1.01 (0.92, 1.10)	0.913
Risk Score 1b	0.99 (0.91, 1.08)	0.835	1.00 (0.92, 1.08)	0.903
Risk Score 2a	1.02 (0.94, 1.12)	0.622	1.03 (0.94, 1.12)	0.557
Risk Score 2b	0.97 (0.90, 1.05)	0.477	0.97 (0.90, 1.06)	0.519
Combined Unweighted	1.00 (0.94- 1.07)	0.890	1.01 (0.95- 1.07)	0.801
Combined Weighted	0.98 (0.93- 1.04)	0.502	0.98 (0.93- 1.04)	0.560

Analysis was performed using Cox proportional hazards regression. Model 1 is adjusted for age, sex, body mass index (BMI), smoking, hypertension, cholesterol medication use, and prior cardiovascular disease; Model 2 is adjusted for age, sex, BMI, smoking, hypertension,

cholesterol medication use, prior cardiovascular disease, oral T2D medication use, and insulin use. The hazard ratio (HR) and its 95% confidence interval (CI) are reported.
