# Supplementary material for: Impact of HDL genetic risk scores on coronary artery calcified plaque and mortality in individuals with type 2 diabetes from the Diabetes Heart Study
Source: Cardiovasc Diabetol. 2013 Jun 25;12:95. doi: 10.1186/1475-2840-12-95 (PMC3695806; doi:10.1186/1475-2840-12-95)
Supplement: Additional file 3 — Association between HDL genetic risk score tertiles and all-cause and CVD- mortality using unadjusted proportional hazards regression models and between risk scores and HDL levels using unadjusted marginal models incorporating generalized estimating equations. Hazard ratios (HR) or β estimates (as appropriate) and 95% confidence intervals (CI) are reported relative to the lowest tertile. [file 1475-2840-12-95-S3.pdf]

Additional file 3: Association between HDL genetic risk score tertiles and all-cause and CVD- mortality using unadjusted proportional hazards regression models and between risk scores and HDL levels using unadjusted marginal models incorporating generalized estimating equations. Hazard ratios (HR) or β estimates (as appropriate) and 95% confidence intervals (CI) are reported relative to the lowest tertile.

			**HDL**		**All-cause mortality**		**CVD mortality**	
	**Tertiles**	**Tertile Ranges**	**β Estimate (95% CI)**	**p-value**	**HR (95% CI)**	**p-value**	**HR (95% CI)**	**p-value**
**Risk Score 1a**	T1	≤ 13	0		1		1	
	T2	14-15	-0.012 (-0.153,0.129)	0.869	1.06 (0.77, 1.47)	0.719	1.21 (0.73, 2.01)	0.451
	T3	≥ 16	0.178 (0.027, 0.328)	0.021	0.98 (0.70, 1.38)	0.908	1.29 (0.76, 2.20)	0.350
**Risk Score 1b**	T1	≤ 12	0		1		1	
	T2	13-14	-0.034 (-0.167, 0.100)	0.622	1.12 (0.83, 1.52)	0.449	1.29 (0.83, 2.01)	0.261
	T3	≥ 15	0.171 (0.021, 0.321)	0.025	0.95 (0.68, 1.33)	0.761	1.20 (0.72, 2.00)	0.490
**Risk Score 2a**	T1	≤ 12	0		1		1	
	T2	13-14	0.083 (-0.047, 0.213)	0.210	0.81 (0.59, 1.10)	0.172	0.86 (0.55, 1.36)	0.529
	T3	≥ 15	0.213 (0.061, 0.366)	0.006	0.84 (0.60, 1.17)	0.295	1.08 (0.68, 1.72)	0.740
**Risk Score 2b**	T1	≤ 10	0		1		1	
	T2	11-12	0.129 (0.003, 0.254)	0.045	0.74 (0.56, 0.98)	0.038	0.81 (0.55, 1.20)	0.292
	T3	≥ 13	0.319 (0.166, 0.471)	4.31×10^{-5}	0.57 (0.39 ,0.83)	0.003	0.72 (0.43, 1.19)	0.199
